# Environmental Gradients and Hen Spatial Distribution in a Cage-Free Aviary System: Internet of Things-Based Real-Time Monitoring for Proactive Management

**DOI:** 10.3390/ani15091225

**Published:** 2025-04-26

**Authors:** Francesco Bordignon, Mattia Pravato, Angela Trocino, Gerolamo Xiccato, Francesco Marinello, Andrea Pezzuolo

**Affiliations:** 1 Department of Agronomy, Food, Natural Resources, Animals and Environment (DAFNAE), University of Padova, 35020 Legnaro, Padova, Italy; francesco.bordignon@unipd.it (F.B.); mattia.pravato@phd.unipd.it (M.P.); angela.trocino@unipd.it (A.T.); gerolamo.xiccato@unipd.it (G.X.); 2 Department of Comparative Biomedicine and Food Science (BCA), University of Padova, 35020 Legnaro, Padova, Italy; 3 Department of Land, Environment, Agriculture and Forestry (TESAF), University of Padova, 35020 Legnaro, Padova, Italy; francesco.marinello@unipd.it

**Keywords:** precision livestock farming, internet of things, IoT, laying hens, particulate matter, NH_3_, CO_2_, flock noise

## Abstract

This study assessed environmental gradients at different heights in a multi-tiered aviary and their relationship with hen spatial distribution. Two pens of an experimental aviary housing 225 Novogen hens (Novogen S.A.S., Plédran, France) each were monitored when their hens were 32–52 weeks old over three periods (January, March, and June). The environmental conditions (humidity, sound intensity, NH_3_, CO_2_, and particulate matter; PM: PM_1_, PM_2.5_, PM_4_, and PM_10_) were continuously recorded every 10 min, 24 h per day, at three heights (floor and middle and upper tiers) using an IoT-based system. Hen distribution was analysed using video recordings from 04:00 to 20:00, scanning 15 min for every 30 min of video. On the whole, the air quality was always consistent with the recommended values for laying hens. The middle tier, where nests were located, exhibited a higher humidity and CO_2_ and PM concentration compared to the upper tier and floor, suggesting reduced airflow. This result can be related to the reduced length of the experimental barn containing only one row of equipment, which likely affected air circulation and distribution compared to what happens in commercial barns with several rows and a length over 50 m. Hen distribution varied during the day, with hen presence on the floor being highest in the midday (58% of hens) and correlated with increased particulate matter (r = 0.57–0.60; *p* < 0.001) and NH_3_ concentrations (r = 0.33; *p* < 0.001). Hens occupied the upper tier more in June (34% vs. 24% in January; *p* < 0.001), correlating with lower humidity and PM levels. Understanding daily and seasonal changes in environmental gradients at different aviary tiers could help optimise ventilation schemes and air quality control and ensure hen welfare, health, and production throughout the laying cycle.

## Simple Summary

This research aimed to investigate how environmental factors, like humidity, gases, and airborne pollutants, vary at different heights in a multi-tiered aviary system and how these changes relate to hen spatial distribution. This study also examined how integrating real-time environmental monitoring with preset thresholds can enable proactive interventions to optimise hen health and welfare in aviary systems. The environmental conditions recorded during the trial were always in line with the recommended values for laying hens. Within the small experimental barn used in the present study, at the middle tier, the results showed higher humidity and particulate matter levels compared to the upper tier and the floor. Hens preferred the upper tier at night and the floor during midday and the afternoon. Seasonal changes also influenced hen distribution, with more hens occupying the upper tier in warmer months, likely due to increased ventilation. A strong positive correlation was found between hen presence and the concentration of particulate matter and ammonia on the floor. These findings emphasise the need to understand environmental gradients within aviaries to optimise air quality control. By addressing the specific environmental conditions of different aviary zones, farmers and the poultry industry can enhance the design and management of aviaries to promote hen health, welfare, and productivity.

## 1. Introduction

The egg industry has undergone substantial changes in recent decades, transitioning from conventional cage housing to cage-free systems [1]. In this context, aviary systems are increasingly favoured as they provide laying hens with more space and greater opportunities to express species-specific behaviours [2]. However, their complex multi-tiered structures present management challenges to ensure optimal environmental conditions, as well as flock health and welfare [3,4,5]. While advancements in sensor technology and data modelling for livestock systems offer promising solutions for the continuous, real-time monitoring of barn conditions, animal health, and welfare [6], their practical integration into egg layer houses remains limited [7,8,9,10].

Environmental monitoring in layer barns has historically focused on conventional cage systems [11,12,13]. Additionally, research has primarily explored horizontal environmental gradients, measured conditions at the manure level, or assessed air quality at exhaust fans [12,14], whereas information on vertical stratification and environmental conditions at the bird level remains limited [15]. Given the complex spatial structure of aviaries, understanding the environmental gradients that could form at different heights and tiers is crucial for improving management strategies.

Traditional environmental monitoring in poultry houses relies on short-term measurements, offering only a limited view of pollution dynamics, potentially missing diurnal or seasonal fluctuations [14,16]. In contrast, an advanced IoT monitoring system enables continuous, high-resolution data collection across multiple parameters and could provide a dynamic and comprehensive assessment of the barn environment [17]. This real-time approach is particularly beneficial in cage-free systems, where environmental conditions could fluctuate throughout the day due to hen spatial distribution and interactions with different areas of the aviary.

In cage-free systems, hens move throughout the day, exhibiting either a typical flock synchrony [18,19] or tending to distribute evenly within the system, depending on the specific design of the aviary and on resource availability [20], as well as the specific design and air control system of the barn. Previous studies on hen spatial distribution in aviaries were conducted either in systems where movement was restricted in the morning and hens were prevented from accessing the litter area until the afternoon [1,19] or in perch-based systems with no tiers [18]. Consequently, little is known about the relationships between hen spatial distribution and the environmental gradients created within the aviary system.

Therefore, the present study aimed to monitor the environmental conditions that form at different heights within a multi-tiered aviary system to provide a more comprehensive understanding of the potential microenvironments experienced by hens. Moreover, relationships between the hen spatial distribution within the aviary and its environmental conditions were explored. Finally, this study explored the integration of real-time monitoring with preset thresholds to enable proactive interventions for the optimisation of aviary environmental conditions.

## 2. Materials and Methods

### 2.1. Experimental Facility, Animals, and Arrangement

**Figure 1:**
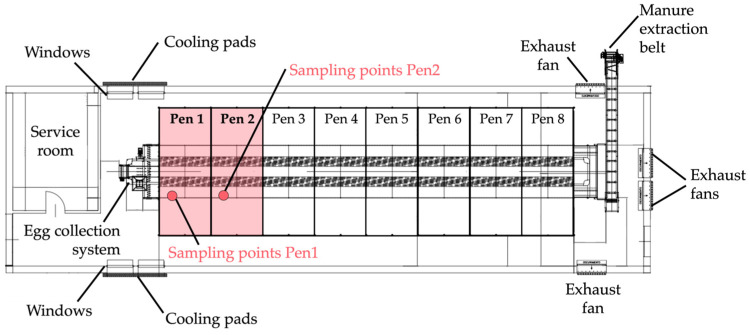
Schematic layout of hen barn and aviary system and sampling points for environmental monitoring.

**Figure 2:**
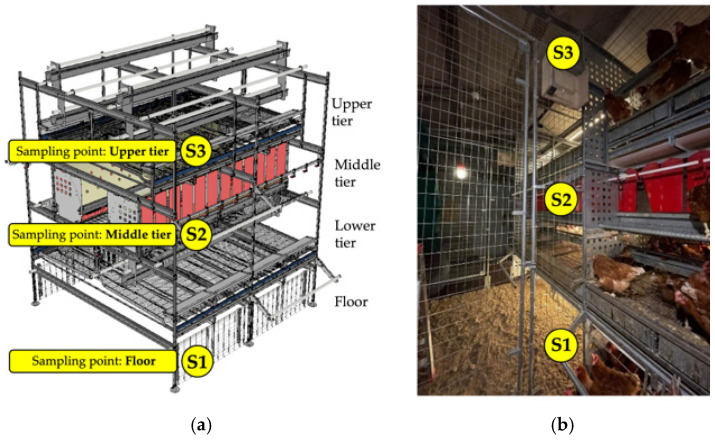
Design of a single module of the aviary system (one module per pen) (**a**) and details of the vertical sampling points within the pen for the monitoring of its environmental conditions (**a**,**b**).

The experiment was conducted at the “L. Toniolo” experimental farm of the University of Padua. The experimental aviary (Figure 1) measured 2.50 m in width × 19.52 m in length × 2.83 m in height and included three tiers and a litter area on the ground. Two side corridors were available for the hens, resulting in a total floor space of 6.01 m × 19.52 m. The aviary was divided into eight pens (2.50 m × 2.44 m × 2.83 m each) (Figure 1), housing 225 hens per pen at 18 weeks of age, corresponding to a stocking density of 9 hens/m^2^ of available space (including tiers). Each pen was equipped with four collective nests (one nest per 55–60 birds; nest area: 0.51 m^2^) (Figure 2). The environmental systems, feeding and drinking lines, lighting, and ventilation, were controlled using a programmable logic controller (Officine Facco & C. Spa, Campo San Martino, Padova, Italy).

The three tiers of the aviary had different functional setups. The first tier, located at a height of 1 m, was equipped with automatic feeders, nipple drinkers, feeding perches, a faeces collection belt, and external perches with a length of 1.20 m. The second tier, at a height of 1.40 m, was fitted with collective nests and nipple drinkers. The third tier, at a height of 2.40 m, included automatic feeders, continuous perches, feeding perches, a faeces collection belt, and uppermost perches running the full length of the pen (Figure 2).

In total, 1800 Novogen hens (Novogen S.A.S., Plédran, France), half of a brown and half of a white genotype, were housed in the aviary (4 pens with brown and 4 pens with white hens). For this study, two pens were monitored, with one pen assigned to each genotype (white and brown hens) (Figure 1). Animal distribution and behaviour data were recorded between 4:00 am and 8:00 pm across all the aviary tiers, including the ground floor, first tier, second tier, and third tier.

**Figure 3:**
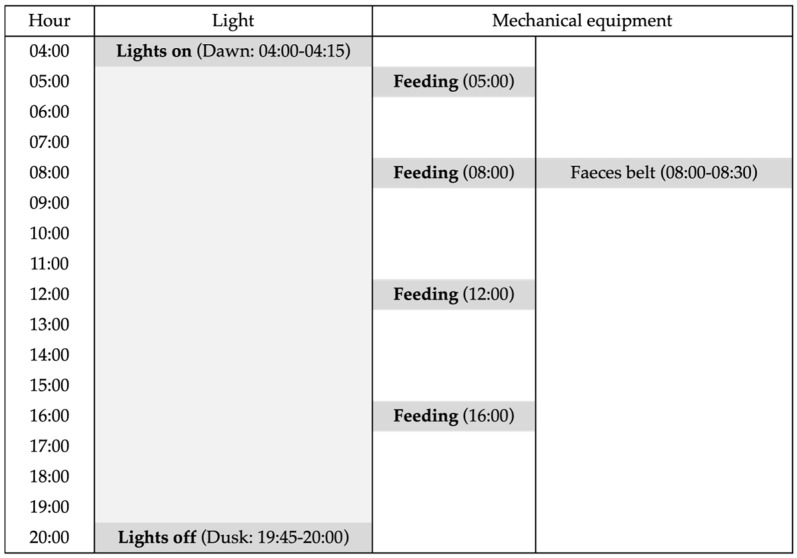
Operating daily schedule for light and mechanical equipment functioning in the aviary.

The data collection for this study was performed over three recording periods (January, March, and June) for 3 weeks each, with hens aged 31–33 weeks (January), 37–40 weeks (March), and 50–52 weeks (June). During the trial, the barn temperature averaged 19.9 ± 1.7 °C, while the external temperature ranged from a minimum of 0.3 °C in January to a maximum of 26.3 °C in June (Figure S1). The hens were exposed to a photoperiod of 16 h of light (4:00–20:00) and 8 h of dark (Figure 3) and were fed ad libitum with a commercial maize and soybean-based diet formulated to meet the nutritional requirements of laying hens.

### 2.2. Aviary Management

The aviary system operated on a consistent daily schedule for lighting and mechanical equipment (Figure 3). The lighting regime simulated the natural dawn and dusk, with lights turning on gradually between 04:00 and 04:15 (dawn) and off between 19:45 and 20:00 (dusk), providing 16 h of light per day. Mechanical operations included four feeding cycles and one faeces belt operation per day. Feed distribution automatically started and occurred at 05:00, 08:00, 12:00, and 16:00 h. The faeces belt operated once daily from 08:00 to 08:30, coinciding with the second feed distribution.

### 2.3. Video Recording and Hen Distribution

A video recording system was used to collect data on the spatial distribution of the hens. The system included eight infrared mini-dome cameras (4 MP resolution, 1080p; HAC-HDW1220MP, Zhejiang Dahua Technology Co., Ltd., Hangzhou, China) and two full-HD video recorders (NVR2116HS-4KS2, Zhejiang Dahua Technology Co., Ltd., Hangzhou, China).

Cameras were positioned to capture the hens in all tiers of the aviary. In each pen, one camera was mounted at approximately 3 m to monitor hens on the floor from above. Another camera was installed at 1.50 m to observe hens on the first and second tiers, while a third camera was placed at 2.80 m to monitor hens on the third tier. Additionally, an overhead camera was installed above the third tier in each pen.

The system was programmed to record and store 24 h footage weekly from all cameras. Recordings took place when the lights were on (4:00 to 20:00), following the environmental monitoring schedule, which consisted of three rounds of five consecutive days during each sampling period (January, March, and June).

To assess hen spatial distribution, the number of hens present on each tier—floor, first, middle, and upper tiers—was recorded by a human operator every 30 min by scanning 10 s of video per time point. The data from each tier were then expressed as a percentage of the total number of observed hens.

### 2.4. Environmental Monitoring Through an Integrated Internet of Things System

The environmental variables were recorded using two air quality sensors (N11-Air quality, IBT-Systems, Milano, Italy) specifically designed for applications in livestock housing systems [21]. The sensors measured humidity (%), average sound intensity (dB), and concentrations of carbon dioxide (CO_2_, ppm), ammonia (NH_3_, ppm), and particulate matter (PM_1_, PM_2.5_, PM_4_, PM_10_, μg/m^3^) (Table 1).

**Figure 4:**
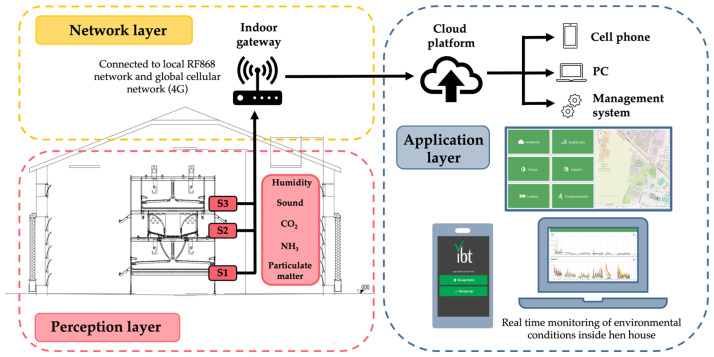
Schematic representation of the IoT-based environmental monitoring system.

The sensors were connected to an IoT-based system (IBT-Systems, Milano, Italy) that comprised three layers: (i) a perception layer with sensors; (ii) a network layer with an indoor gateway connecting to local RF868 and 4G networks; and (iii) an application layer showing cloud-based data processing and real-time monitoring interfaces on various devices (Figure 4).

Given the characteristics of the monitored phenomena, the measurement protocol was designed and detailed as follows:

- Humidity and CO_2_ were measured at one-minute intervals; we collected the data and aggregated them by calculating their mean over each ten-minute window.
- Ammonia (NH_3_) measurements were taken every 10 s, with values averaged across each corresponding 10 min segment.
- Sound pressure: The sensor sampled data every 10 s. For each 10 min interval, both the maximum and average values were computed.
- Particulate matter measurements were taken every 15 min, and the resulting data were transmitted at 10 min intervals. Although this strategy limited the detection of rapid fluctuations, the 15 min sampling rate was adopted to balance measurement fidelity with power efficiency, aiming to enable up to three weeks of uninterrupted operation under battery power.

At every 10 min mark, the firmware aggregated all available sensor data, computing statistical features such as the mean, maximum, and minimum values. These were compiled into a compact, binary-encoded packet, which was subsequently stored in the sensor flash memory and transmitted to the gateway via a proprietary sub-gigahertz wireless link [21].

To capture the environmental conditions at different levels of the aviary, sensors were placed at three specific heights: the ground floor (S1: 0.1 m above the floor), the second tier (S2: 1.8 m above the floor), and the third tier (S3: 2.7 m above the floor) (Figure 2 and Figure 4).

Data were recorded for a period of five consecutive days at each height in the two different pens (Monday to Friday). Since only two sensors were available, after each five-day recording period, the sensors were recharged and relocated to the next sampling height in the same two pens. This process was repeated during the three sampling periods (January, March, and June). Although the environmental data from the three heights of the aviary were collected sequentially rather than simultaneously, this approach was mitigated by conducting the recordings under consistent environmental management. Furthermore, for each season, a high-resolution dataset capable of capturing daily and diurnal variations was collected within a three-week period, preventing large environmental shifts within the hen house.

### 2.5. Statistical Analysis

Statistical analyses were conducted using SAS software (Version 9.4; SAS Institute, Cary, NC, USA) [22]. The distribution data, expressed as the percentage of observed animals, were analysed using a generalised linear mixed model with the PROC GLIMMIX procedure, considering a beta distribution of the data. Fixed effects included the hour of the day, the month of sampling, and their interactions, as well as interactions between the genotype and hour of the day and between the genotype and month of sampling. The pen was included as a random effect.

The environmental condition data were analysed using a mixed model with the PROC GLM procedure. Fixed effects included tier height, hour of the day, month of sampling, and their interactions. Correlations between the hen spatial distribution and environmental conditions at different tiers and on the floor were analysed using the PROC CORR procedure. A univariate logistic regression analysis using PROC LOGISTIC was conducted to assess the effect of the time slot of the day on reaching the PM_2.5_ alarm threshold.

## 3. Results and Discussion

### 3.1. Environmental Conditions

The setup of the experimental aviary and of the barn in which it was located were designed to optimise the management of laying hens and differ from commercial setups, and the environmental conditions recorded during the trial were in line with the recommended values for laying hens (Table 2). Specifically, the humidity remained within the comfort range (50–70%) [23]; CO_2_ concentrations stayed below the maximum threshold (2500 ppm) set by the International Commission of Agricultural and Biosystems Engineering [24] for poultry production; and NH_3_ concentrations consistently remained below the ideal level of 10 ppm [25]. The average barn sound intensity remained below stress-inducing levels for chickens (~90 dB) [26] and aligned with ambient noise levels typically recorded in poultry barns (65–75 dB) [27,28]. Regarding particulate matter, PM_2.5_ concentrations complied with the limit criteria for human health (<75 μg/m^3^) [29] for most of the trial duration, except in January, when they averaged 96.5 μg/m^3^. The low values of particulate matter recorded in our experimental setup contrast with previous results in cage-free aviary systems, which tend to have higher pollutant levels, particularly particulate matter, compared with cage-based systems [30]. In fact, particulate matter concentrations in aviary systems can be up to 7–12 times higher than in conventional cages [30], primarily due to the presence of litter on the floor and increased freedom of movement of the hens, which may resuspend the particulate matter in the litter and create bioaerosols [16,31].

Previous studies have compared gas and particulate matter levels across different housing systems for laying hens under commercial conditions, including colony cages, conventional cages, and aviaries [31,32,33]. However, there is limited information regarding the environmental gradients that may develop within distinct zones of an aviary system. Our results indicate that under our conditions, the middle tier of the aviary could potentially represented a distinct stagnation zone characterised by higher humidity (61.9% vs. 60.3% vs. 57.2%; *p* < 0.001), increased CO_2_ levels (+2.4%; *p* < 0.001) compared to the other tiers, and elevated PM_1_ and PM_2.5_ concentrations, especially when compared with the upper tier (+55% and +31%, respectively; *p* < 0.001). These findings could suggest reduced air circulation in the middle tier, likely due to the presence of the nest line, which may obstruct airflow and create a microenvironment where air pollutants and moisture accumulate [34]. Nevertheless, this result can be related to the reduced length of the experimental barn, containing only one row of equipment, which likely affected air circulation and distribution compared to what happens in commercial barns with more rows and a length above 50 m. Poor air quality and high humidity can increase the risk of respiratory issues [30] and favour the development of noxious airborne bacteria [32]. As for sound levels, the slightly higher noise recorded in the middle tier (68.4 dB vs. 68.0 dB and 67.9 dB; *p* < 0.001) compared with the floor and upper tier, while statistically significant, may not be biologically relevant for the hens, who have shown a stress response when exposed to a sound intensity higher than 95 dB [35,36,37].

The highest NH_3_ concentrations at the floor level are likely a consequence of litter decomposition, which is the primary source of ammonia production [38]. On the other hand, the higher NH_3_ levels in the upper tier compared to the middle tier can be explained by the presence of faeces collection belts in the upper tier, which temporarily retain excreta before removal, allowing for continued ammonia volatilisation. Conversely, the middle tier, housing the nests, lacked a direct faeces collection mechanism; droppings from this level fell to the tier below, potentially reducing localised ammonia accumulation.

In terms of seasonal variations within the aviary, the observed increase in humidity, peaking in June (+58% compared with January; *p* < 0.001), aligns with expected outdoor climate patterns.

**Figure 5:**
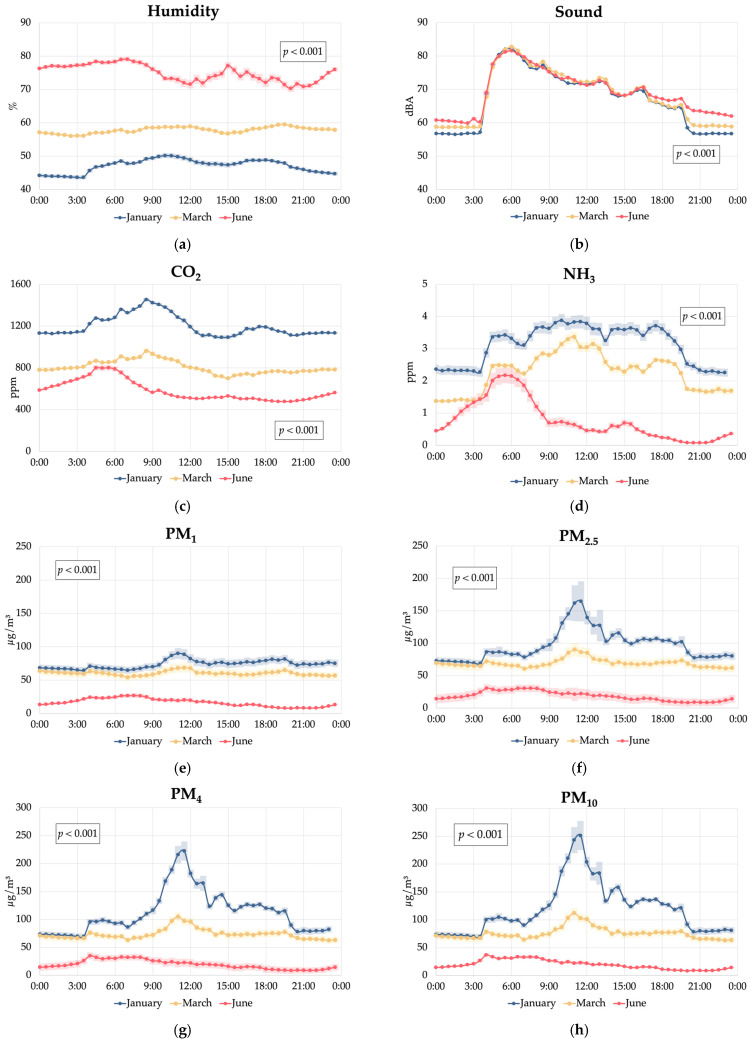
Effect of the interaction between the hour of the day and the sampling cycle (January, March, and June) on the environmental conditions in a cage-free multi-tiered aviary system: (**a**) humidity; (**b**) sound; (**c**) carbon dioxide (CO_2_); (**d**) ammonia (NH_3_); (**e**) particulate matter PM_1_; (**f**) particulate matter PM_2.5_; (**g**) particulate matter PM_4_; and (**h**) particulate matter PM_10_.

Then, the significant increase in sound intensity from January to June (+2.8%; *p* < 0.001) could be related to increased hen activity during longer outdoor daylight hours as well as the higher light intensity, which seemed to be perceived by the hens even though the light schedule within the aviary remained unchanged. This is corroborated by the trend observed in Figure 5, where sound intensity in the barn remained higher in June at night from 18:00 to 4:00 and especially from 18:00 to 0:00 compared with January and March. This could potentially be linked to an apparent sensitivity of hens to seasonal changes in day length, even within a controlled lighting environment, which could have important implications for aviary management and hen welfare. This suggests that hens may respond to subtle environmental cues beyond an artificial lighting schedule [39], possibly due to small amounts of natural light entering the aviary or other seasonal factors that require further investigation. On the other hand, the peak in sound intensity recorded during the early hours of the day could be linked to an increase in hen vocalisations, which have been observed during the early hours of the day (after the start of the artificial lights) both in cage- and perch-based aviary systems [40].

A marked reduction in CO_2_ (−51%; *p* < 0.001), NH_3_ (−76%; *p* < 0.001) and particulate matter (from −84% to −79%; *p* < 0.001) concentrations was found in June compared with January. This seasonal variation in air quality parameters can be primarily attributed to increased ventilation rates during summer [11]. In fact, the ventilation rate set in our aviary controller changed from a minimum of 2.96 m^3^/h per hen in January to a minimum ventilation rate of 6.89 m^3^/h per hen in June. This increased air exchange not only helped regulate temperature but also effectively removed accumulated air pollutants from the aviary environment. Under our optimised conditions, the NH_3_ concentrations measured in January (3.12 ppm) remained substantially below the values observed in previous studies in broiler barns and cage and perchery layer barns in the UK (12.3–24.2 ppm) [41], in conventional cage-based layer barns in Turkey (5.42–8.05 ppm) [13], and in commercial cage-free houses in China (20 ppm) [42]. Our results were also similar to those recorded in conventional cage-based barns in Italy during the summer months (2 ppm) [43]. Similarly, the highest CO_2_ level recorded in January in our study (1200 ppm) was lower than those previously recorded in winter in commercial cage-based barns (1800 to 3072 ppm) [44,45].

As for particulate matter, the apparently high concentrations we recorded in January were substantially lower than those reported in previous studies in commercial cage-free systems (i.e., PM_1_ 400–4250 μg/m^3^; PM_2.5_ 420–4490 μg/m^3^; PM_4_ 500–5250 μg/m^3^; and PM_10_: 381–10,820 μg/m^3^) [30,31,32,43], as well as in cage systems (PM_10_: 546 μg/m^3^) [12]. The values in our experimental setup were similar to those found in an experimental aviary system with pullets reared from 1 to 16 weeks of age [15] but were slightly higher than those recorded in commercial conventional-cage and enriched-cage systems (PM_2.5_: 35 μg/m^3^ and 56 μg/m^3^, respectively) [31].

### 3.2. Hen Spatial Distribution

**Figure 6:**
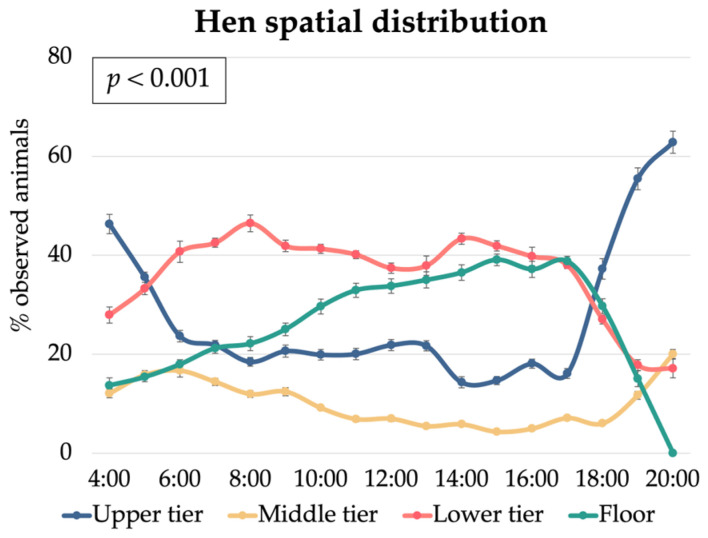
Effect of the hour of day on the laying hen spatial distribution (% of observed animals) on the different tiers (upper tier, middle tier, and lower tier) and floor of the multi-tiered aviary system.

The distribution of the laying hens across the different tiers of the cage-free aviary system varied significantly throughout the day (*p* < 0.001) (Figure 6).

The upper tier was highly populated at the time the lights switched on, with approximately 45% of the hens, as observed at 4:00. However, occupancy declined sharply afterward, reaching its lowest point (about 15%) around 14:00. From 16:00 onwards, a notable increase in upper tier usage was observed, peaking at nearly 65% by 20:00. In contrast, the lower tier exhibited an opposite trend, with the number of hens gradually increasing between 4:00 and 6:00 (from 30% to 48%), reaching its peak at 8:00 (58%). Occupancy in the lower tier then steadily declined in the afternoon, coinciding with the rise in upper tier usage.

The middle tier consistently had the lowest occupancy throughout the day, generally remaining below 20%. While it showed slight fluctuations, its usage was relatively stable compared to the other tiers. Notably, occupancy peaked between 5:00 and 7:00, coinciding with the peak in egg-laying activity, as observed in previous trials in the same aviary [46]. The middle tier had no feeders or drinkers and was primarily used as an access point to the nests, which explains its limited use for other activities. However, the secondary peak in the evening suggests that hens used this tier as an intermediate stop before moving to the upper tier for roosting.

The floor area showed a gradual increase in hen presence from morning to afternoon, reaching approximately 40% occupancy around 16:00. However, usage dropped sharply in the evening, nearing zero by 20:00.

These trends align with the typical circadian behaviour of hens in cage-free aviaries. In the morning, hens prioritise the areas where nests are located to express their laying behaviour [47]. Later in the day, they engage in foraging and dustbathing on the floor and lower tiers [1]. By evening, hens transition to the upper tiers for roosting, a behaviour commonly observed in aviary systems [19].

During daylight hours, the hens displayed a more even distribution across the lower tier and floor, likely related to their feeding, foraging, and other diurnal activities [19,20].

### 3.3. Relationships Between Hen Spatial Distribution and Environmental Conditions

To further understand how environmental variables relate to hen spatial distribution, the correlations between air quality and spatial distribution were analysed.

**Figure 7:**
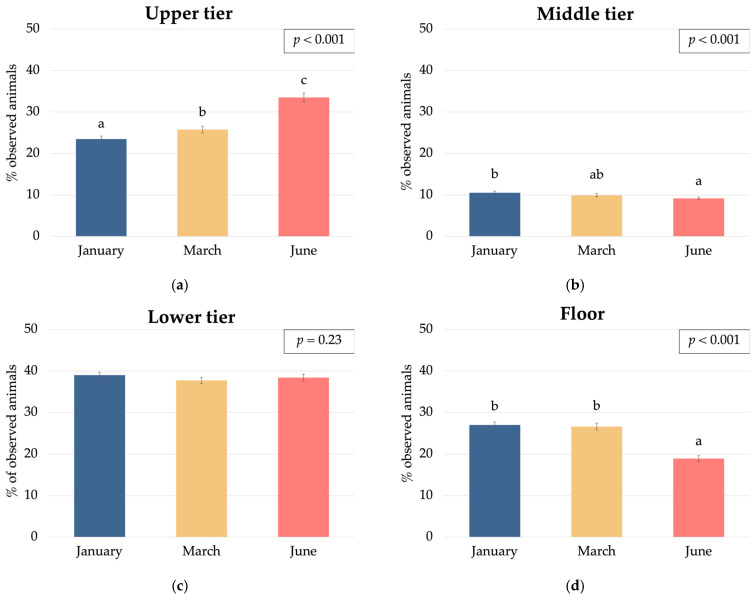
Effect of the month of sampling on laying hen distribution (% of observed animals) within a multi-tiered aviary system: (**a**) upper tier; (**b**) middle tier; (**c**) lower tier; and (**d**) floor. Different letters above bars indicate statistically significant differences between means (*p* < 0.05).

The observed vertical shift in hen distribution from winter to summer, characterised by increased occupancy of the upper tier from January to June (24% in January to 26% in March to 34% in June; *p* < 0.001) and decreased floor occupancy (27% in January/March to 19% in June; *p* < 0.001) (Figure 7), suggests a potential relationship between the specific barn ventilation design and resulting microclimate variations. We hypothesise that the location of cooled air inlets at 2.00 m and 2.40 m of height could have created an airflow pattern that enhanced the cool air circulation/flux in the upper tiers (2.40 m height) compared to the floor level.

**Figure 8:**
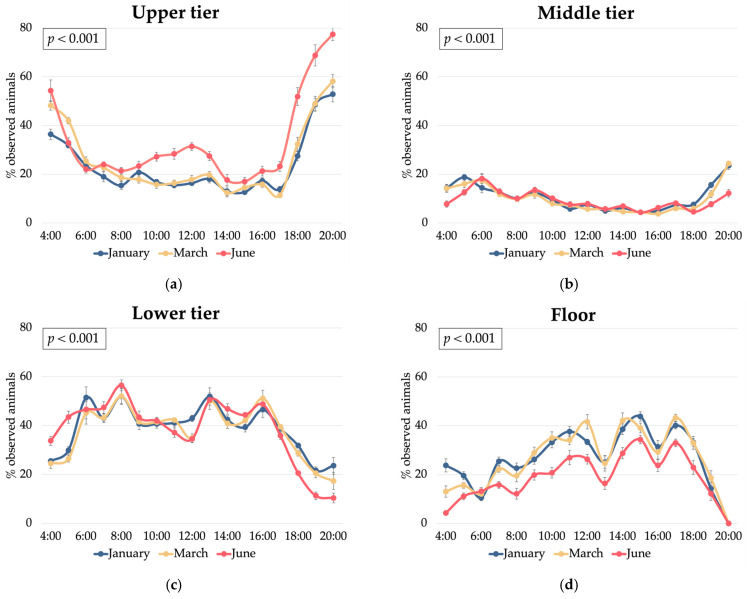
Effect of the interaction between the hour of day and the month of sampling on laying hen distribution (% of observed animals) within a multi-tiered aviary system: (**a**) upper tier; (**b**) middle tier; (**c**) lower tier; and (**d**) floor.

**Figure A1:**
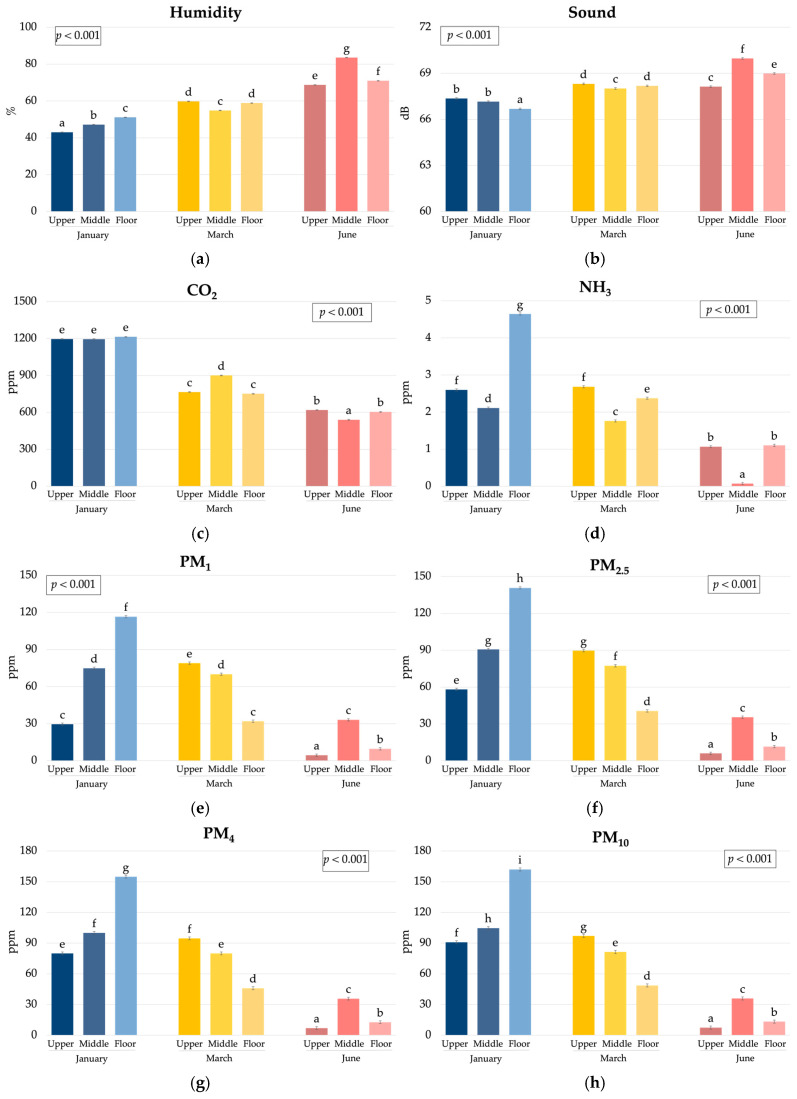
Effect of the interaction between the tier height (upper tier, middle tier, and floor) and the month of sampling on the environmental conditions in a cage-free multi-tiered aviary system: (**a**) humidity; (**b**) sound; (**c**) carbon dioxide (CO_2_); (**d**) ammonia (NH_3_); (**e**) particulate matter PM_1_; (**f**) particulate matter PM_2.5_; (**g**) particulate matter PM_4_; and (**h**) particulate matter PM_10_. Different letters above bars indicate statistically significant differences between means (*p* < 0.05).

This fact could have potentially resulted in a more favourable microclimate in the upper tier during the summer months, attracting a higher proportion of hens to this area, and also during the central hours of the day (Figure 8a), when they usually tend to dustbathe and forage in the litter [19]. The lower humidity and PM concentrations in the upper tier in June compared to the floor and middle tier (Figure A1) support this hypothesis despite the higher hen occupancy, which needs validation. Specifically, accurate measurements of airflow patterns throughout the aviary system are required to establish direct relationships between the ventilation dynamics, hen spatial distribution, and environmental conditions at different tiers.

**Figure 9:**
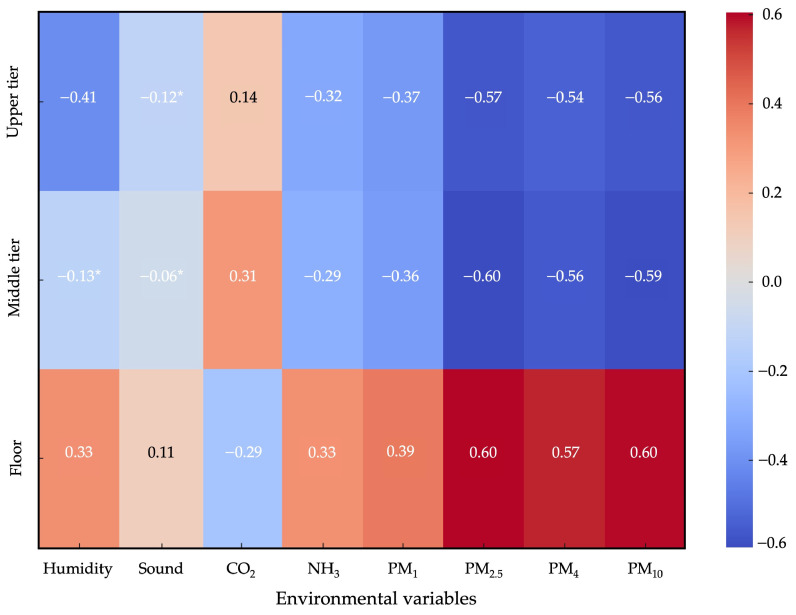
Correlation between the hen spatial distribution (% of observed hens) and environmental variables at different tiers (upper tier, middle tier) and on the floor of a cage-free aviary system. * *p* > 0.05.

**Figure 10:**
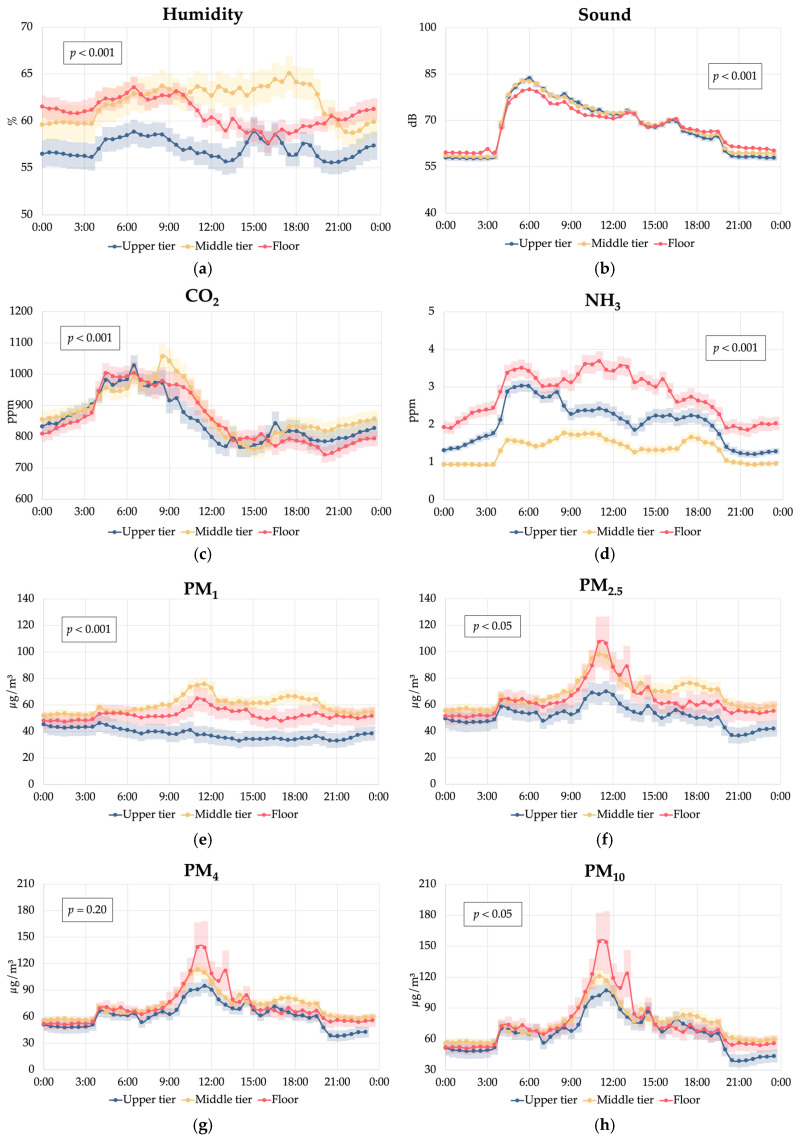
Effect of the interaction between the hour of the day and the tier height (upper tier, middle tier, or floor) on the environmental conditions in a cage-free multi-tiered aviary system: (**a**) humidity; (**b**) sound; (**c**) carbon dioxide (CO_2_); (**d**) ammonia (NH_3_); (**e**) particulate matter PM_1_; (**f**) particulate matter PM_2.5_; (**g**) particulate matter PM_4_; and (**h**) particulate matter PM_10_.

Significant correlations between the distribution of hens across different tiers of the aviary and the environmental conditions were observed (Figure 9). As expected, particulate matter concentrations were positively correlated with hen presence on the floor (r = 0.57–0.60; *p* < 0.001), confirming that hen activity on the floor (i.e., with dustbathing, scratching, and foraging behaviours) is the primary driver of airborne particulate matter in the aviary [33]. This could explain the peaks in PM concentrations on the floor observed in the aviary during the central hours of the day (Figure 10), when hens preferred to stay on the floor rather than on the middle and upper tiers (Figure 6). The negative correlations (−54 < r < −60; *p* < 0.001) found in the upper tiers could suggest that these areas showed reduced PM levels regardless of the high presence of hens, likely due to the physical distance from the main dust source on the floor.

The changes in ammonia concentrations were consistent with those described for particulate matter, being positively correlated with hen presence on the floor (Figure 8 and Figure 9) and negatively correlated with hen presence in the upper tiers. This pattern is likely due to the increased ammonia production and volatilisation from fresh faeces when hens occupied the floor [38,41]. Additionally, the presence of hens may elevate local temperature and humidity levels through respiration and excretion, further promoting NH_3_ release [48]. Another hypothesis could be related to the density differences between ammonia and carbon dioxide. Ammonia, being lighter than air (0.73 kg/m^3^ vs. 1.23 kg/m^3^), tends to accumulate in the upper layers under calm air conditions, whereas increased turbulence caused by hen activity during evening hours in the upper tiers could likely contributed to its dispersion. Conversely, carbon dioxide (1.98 kg/m^3^), being denser than air, tends to stratify near the ground, with hen movement promoting air mixing and consequently reducing its concentration.

Interestingly, higher CO_2_ levels were mildly correlated with increased hen presence in the middle tier (r = 0.33; *p* < 0.001), while showing a negative correlation with floor occupancy. This pattern suggests that CO_2_, primarily generated through hen respiration, accumulated in the middle tier due to the potentially less-efficient air exchange in this area [34], as previously discussed.

The sound levels showed weak correlations across the tiers, indicating relatively uniform noise generation regardless of hen distribution without exhibiting any stratification. However, changes in sound intensity during the day were predominantly determined by the hens’ activity level (Figure 5).

### 3.4. Implications: Real-Time Monitoring for Proactive Interventions

The real-time monitoring data revealed that PM_2.5_ concentrations on the floor frequently exceeded the “WARNING” threshold and occasionally reached the “ALARM” zone, particularly during specific time slots throughout the day. A logistic regression analysis (Table 3) identified the morning (8:00–12:00) and afternoon (12:00–16:00) periods as having significantly higher odds of reaching critical PM_2.5_ levels, with odds ratios of 16.8 (95% CI: 3.92–71.7) and 6.41 (95% CI: 1.41–29.1), respectively, compared to the early morning (4:00–8:00) reference period.

**Figure 11:**
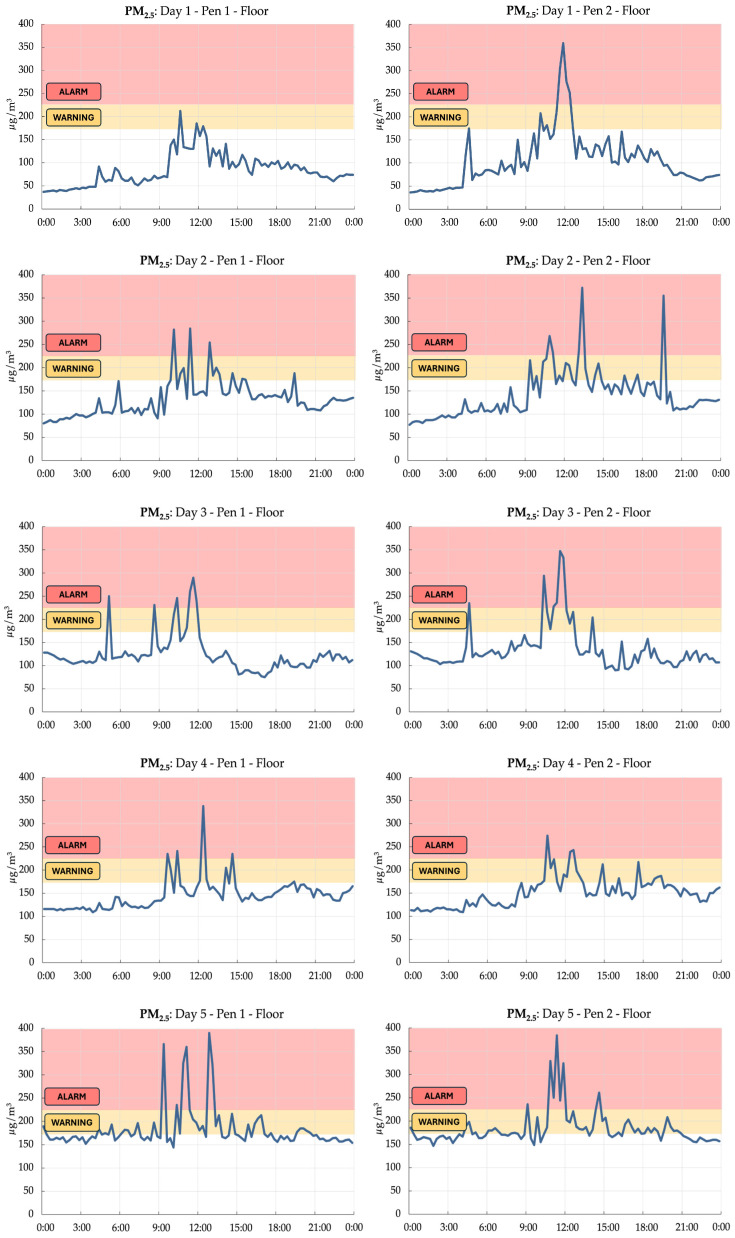
Real-time daily monitoring of PM_2.5_ on the floor during winter and the setting of threshold zones for proactive interventions. Example of five days of recordings.

Focusing on PM_2.5_ levels on the floor during the winter period, which represented the most critical scenario observed in this study, we identified an “ALARM” zone above 230 μg/m^3^, a concentration considered potentially harmful for livestock [49]. Additionally, a “WARNING” zone was established between 180 and 230 μg/m^3^, indicating the need for preventive measures to avoid further PM accumulation (Figure 11).

The continuous, high-resolution monitoring approach employed in this study, leveraging an Internet of Things system, demonstrates the potential for real-time environmental assessment, which could be used for proactive management interventions within cage-free aviary systems. By establishing thresholds for critical environmental parameters, such as for PM concentrations, aviary operators can implement targeted mitigation strategies to maintain optimal conditions for hen health and welfare in addition to farmers’ comfortable working conditions.

These findings highlight the potential for implementing proactive interventions based on real-time monitoring data and predetermined thresholds. For instance, during the identified high-risk time slots (morning and afternoon), aviary operators could initiate targeted ventilation adjustments or litter management practises to mitigate PM accumulation on the floor. Such interventions could include increasing ventilation rates, employing litter amendments, or temporarily restricting hen access to specific areas to minimises litter disturbance. Moreover, the continuous monitoring approach enables the detection of acute PM spikes, which may be missed by intermittent sampling methods.

### 3.5. Limitations

This study was performed in a small-scale experimental barn with an experimental aviary setup. Therefore, the environmental results and gradients observed under our conditions may not directly reflect those of large, cage-free aviary facilities, which can exhibit substantial variations in design, configuration, and management practises. Further validation in commercial-scale facilities is recommended to assess the system’s performance under operational conditions, to determine its scalability and applicability across different commercial contexts, and to confirm whether the patterns and changes in environmental conditions observed in this study are consistent in larger commercial settings.

Monitoring was conducted only in two pens located in the first section of the aviary. While this enabled an in-depth analysis of vertical environmental gradients, it did not account for potential horizontal variations across different sections of the aviary. Expanding the monitoring to include pens in the middle and final sections would provide further insights into the spatial heterogeneity of the environmental conditions throughout the aviary system. Additionally, due to the need for periodic sensor recharging (approximately every 48 h), environmental data collection was conducted in five-day slots rather than continuously. Future studies should aim for uninterrupted data collection and optimise sensor battery life to capture a more comprehensive picture of the environmental dynamics within the aviary system.

## 4. Conclusions

This study highlights the presence of environmental gradients within a multi-tiered aviary, with the middle tier exhibiting higher humidity and CO_2_ and particulate matter concentrations, likely due to the reduced airflow under the specific experimental setup of the present study.

Hen spatial distribution followed a distinct daily pattern, with upper-tier occupancy peaking at night and floor use being highest at midday. Seasonal shifts in their spatial distribution were also observed, with increased upper-tier occupancy in summer, possibly due to improved ventilation and microclimate conditions. The strong correlations between hen presence and air quality parameters suggest that hen activity on the floor contributes to particulate matter and ammonia accumulation, while CO_2_ tends to concentrate in the middle tier.

These findings emphasise the need to measure environmental gradients in the three-dimensional space of aviary systems to optimise ventilation design under each specific working condition to mitigate localised air quality issues and improve hen welfare. Additionally, the integration of both real-time environmental monitoring and predetermined thresholds could enable proactive interventions to manage air quality and maintain optimal conditions throughout the farming cycle.

## Figures and Tables

**Table 1:** Characteristics of the sensors integrated in the IoT system.

Node	Sensor	Technology	Measurement	Range	Accuracy
N1	Sensirion SHT30	CMOSens	Temperature	−10–+60 °C	±0.2 °C
	Sensirion SHT30	CMOSens	Relative humidity	0–100%	±2%
	SPW2430	MEMS	Sound intensity	30–130 dBA	±2%
N2	Sensirion SCD30	Nondispersive infrared (NDIR)	CO_2_ concentration	400–10,000 ppm	±3%
	GS +4NH3100	Electrochemical cell	NH_3_ concentration	0–100 ppm	±10%
N3	-	Laser scattering	PM_1_, PM_2.5_, PM_4_, PM_10_	0–1000 μg/m^3^	±10 μg/m^3^ ±25 μg/m^3^

**Table 2:** Differences in the environmental conditions in a cage-free multi-tiered aviary system for laying hens according to the tier of the aviary and the month of sampling.

Variables	Tier (T)			Month (M)			*p*-Value						RMSE
	Floor	Middle	Upper	January	March	June	Hour (H)	T	M	H × T	H × M	T × M	
Humidity, %	60.3 ^b^	61.9 ^c^	57.2 ^a^	47.1 ^a^	57.8 ^b^	74.4 ^c^	<0.001	<0.001	<0.001	<0.001	<0.001	<0.001	5.12
Sound, dB	68.0 ^b^	68.4 ^c^	67.9 ^a^	67.1 ^a^	68.2 ^b^	69.0 ^c^	<0.001	<0.001	<0.001	<0.001	<0.001	<0.001	2.01
CO_2_, ppm	856 ^a^	878 ^b^	860 ^a^	1201 ^c^	806 ^b^	587 ^a^	<0.001	<0.001	<0.001	<0.001	<0.001	<0.001	130
NH_3_, ppm	2.71 ^c^	1.31 ^a^	2.12 ^b^	3.12 ^c^	2.27 ^b^	0.75 ^a^	<0.001	<0.001	<0.001	<0.001	<0.001	<0.001	1.04
PM_1_, μg/m^3^	52.1 ^b^	59.3 ^c^	38.3 ^a^	73.6 ^c^	60.3 ^b^	15.7 ^a^	<0.001	<0.001	<0.001	<0.001	<0.001	<0.001	29.4
PM_2.5_, μg/m^3^	63.6 ^b^	67.8 ^c^	51.9 ^a^	96.5 ^c^	69.2 ^b^	17.7 ^a^	<0.001	<0.001	<0.001	<0.001	<0.001	<0.001	35.3
PM_4_, μg/m^3^	70.5 ^b^	71.9 ^b^	61.2 ^a^	112 ^c^	73.5 ^b^	18.5 ^a^	<0.001	<0.001	<0.001	<0.001	<0.001	<0.001	42.9
PM_10_, μg/m^3^	74.0 ^b^	74.0 ^b^	65.8 ^a^	119 ^c^	75.7 ^b^	18.9 ^a^	<0.001	<0.001	<0.001	<0.001	<0.001	<0.001	47.5

RMSE: Root mean square error. Data are expressed as LS means. ^a,b,c^ Different superscript letters above means indicate significant differences.

**Table 3:** Logistic regression analysis of the odds of reaching the “ALARM” zone (above 230 μg/m^3^) [49] for PM_2.5_ concentrations on the floor over different time slots.

Variable	Estimate	SE	Odds Ratio	95% CI		*p*-Value
				Lower 95%	Upper 95%	
Intercept	−4.37	0.71	-	-	-	<0.001
Time period						
Early (4:00–8:00)	-	-	-	-	-	-
Morning (8:00–12:00)	2.82	0.74	16.8	3.92	71.7	<0.001
Afternoon (12:00–16:00)	1.86	0.77	6.41	1.41	29.1	<0.05
Late (16:00–20:00)	−0.76	1.23	0.47	0.04	5.21	0.54

## Data Availability

The original contributions presented in this study are included in the article; further inquiries can be directed to the corresponding author.

## Supplementary Materials

The following supporting information can be downloaded at: https://www.mdpi.com/article/10.3390/ani15091225/s1.

## Abbreviations

The following abbreviations are used in this manuscript:

PM	Particulate matter
RMSE	Root mean square error
