# Supplementary figures and images for: Environmental Gradients and Hen Spatial Distribution in a Cage-Free Aviary System: Internet of Things-Based Real-Time Monitoring for Proactive Management
Source: Animals (Basel). 2025 Apr 26;15(9):1225. doi: 10.3390/ani15091225 (PMC12070870; doi:10.3390/ani15091225)

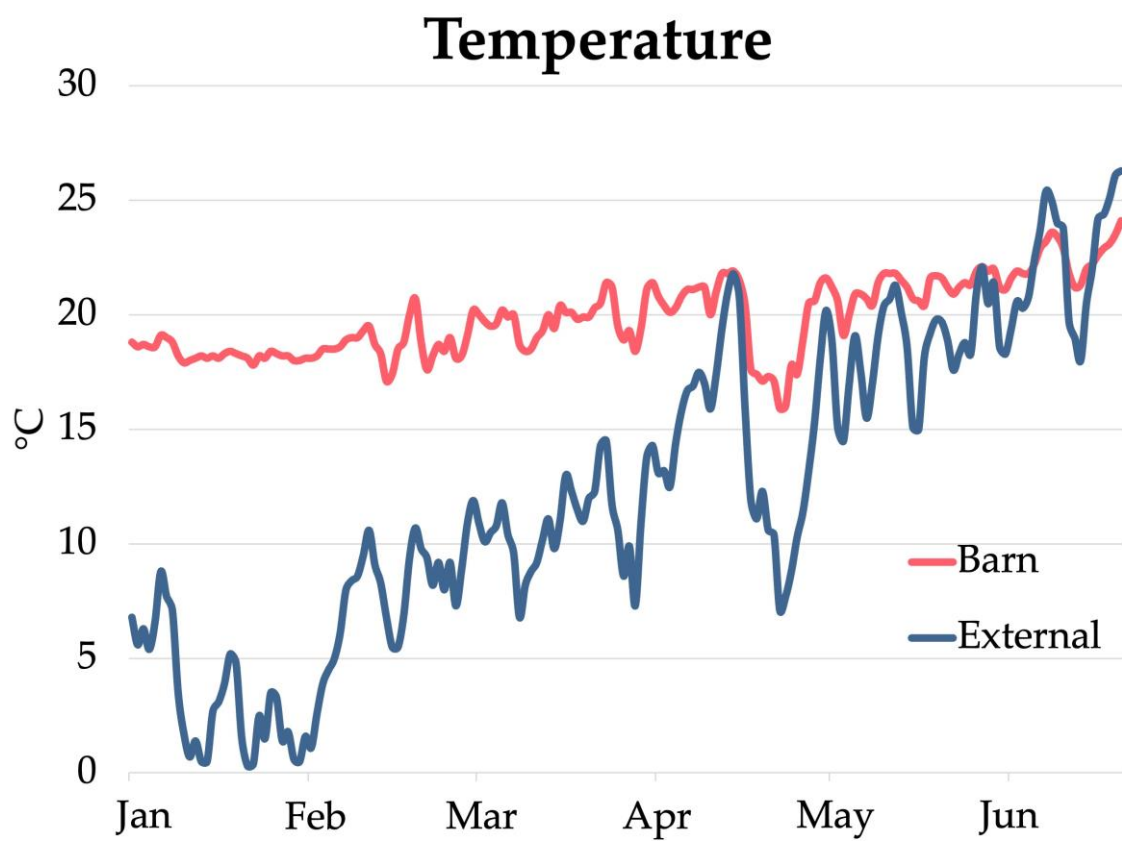

**Figure S1.** Changes in internal (barn) and external temperatures recorded during the trial.

Supplement: Supplementary file 1 [file animals-15-01225-s001.zip › animals-3571949-supplementary.pdf]
